# *MIR27A* Gene Polymorphism Modifies the Effect of Common *DPYD* Gene Variants on Severe Toxicity in Patients with Gastrointestinal Tumors Treated with Fluoropyrimidine-Based Anticancer Therapy

**DOI:** 10.3390/ijms25158503

**Published:** 2024-08-04

**Authors:** Anna Ikonnikova, Denis Fedorinov, Dmitry Gryadunov, Rustam Heydarov, Marina Lyadova, Alexey Moskalenko, Vladimir Mikhailovich, Marina Emelyanova, Vladimir Lyadov

**Affiliations:** 1 Center for Precision Genome Editing and Genetic Technologies for Biomedicine, Engelhardt Institute of Molecular Biology, Russian Academy of Sciences, 119991 Moscow, Russia; grad@biochip.ru (D.G.); emel.marina85@gmail.com (M.E.); 2 Oncology Center No. 1, Moscow City Hospital Named after S. S. Yudin, Moscow Healthcare Department, 117152 Moscow, Russia; fedorinov.denis@gmail.com (D.F.); dr.lyadova@gmail.com (M.L.); mansurgkokod@gmail.com (A.M.); vlyadov@gmail.com (V.L.); 3 Department of Oncology and Palliative Medicine Named after Academician A.I. Savitsky, Russian Medical Academy of Continuous Professional Education, 123242 Moscow, Russia; 4 Laboratory of Biological Microchips, Engelhardt Institute of Molecular Biology, Russian Academy of Sciences, 119991 Moscow, Russia; rustam.heydarov@gmail.com (R.H.); v.mikhailovich@gmail.com (V.M.); 5 Department of Oncology, Novokuznetsk State Institute for Postgraduate Medical Education, 654005 Novokuznetsk, Russia

**Keywords:** pharmacogenetics, *DPYD*, *MIR27A*, fluoropyrimidines, toxicity, chemotherapy, dihydropyrimidine dehydrogenase, miR-27a, personalized medicine, oncology

## Abstract

To reduce severe fluoropyrimidine-related toxicity, pharmacogenetic guidelines recommend a dose reduction for carriers of four high-risk variants in the *DPYD* gene (*2A, *13, c.2846A>T, HapB3). The polymorphism in the *MIR27A* gene has been shown to enhance the predictive value of these variants. Our study aimed to explore whether rs895819 in the *MIR27A* gene modifies the effect of five common *DPYD* variants: c.1129-5923C>G (rs75017182, HapB3), c.2194G>A (rs1801160, *6), c.1601G>A (rs1801158, *4), c.496A>G (rs2297595), and c.85T>C (rs1801265, *9A). The study included 370 Caucasian patients with gastrointestinal tumors who received fluoropyrimidine-containing chemotherapy. Genotyping was performed using high-resolution melting analysis. The DPYD*6 allele was associated with overall severe toxicity and neutropenia with an increased risk particularly pronounced in patients carrying the *MIR27A* variant. All carriers of DPYD*6 exhibited an association with asthenia regardless of their *MIR27A* status. The increased risk of neutropenia in patients with c.496G was only evident in those co-carrying the *MIR27A* variant. DPYD*4 was also significantly linked to neutropenia risk in co-carriers of the *MIR27A* variant. Thus, we have demonstrated the predictive value of the *6, *4, and c.496G alleles of the *DPYD* gene, considering the modifying effect of the *MIR27A* polymorphism.

## 1. Introduction

Fluoropyrimidines (FPs), including 5-fluorouracil and capecitabine, are widely used in the treatment of various solid tumors as part of chemotherapy regimens. Despite their proven efficiency, the use of FPs commonly leads to severe toxicity, followed by interruptions and delays in chemotherapy delivery, life-threatening events and even death [1].

A well-known cause of severe FP-related toxicity is the deficiency of dihydropyrimidine dehydrogenase (DPD) activity as far as DPD plays a crucial role in metabolizing these drugs. Numerous studies have identified four allelic variants in the *DPYD* gene that encode DPD, leading to decreased or absent DPD enzymatic activity, and they are associated with severe FP toxicity with a high level of evidence: *2A (rs3918290), *13 (rs55886062), -2846A>T (rs67376798), and c.1129-5923C>G/HapB3 (rs75017182) [2,3]. Pharmacogenetic guidelines recommend a 25–50% dose reduction or avoidance of FPs for carriers of these variants [2,3,4]. Several prospective studies demonstrated that a genotype-based treatment approach (namely, drug dose reduction in DPD-deficient patients) could drastically reduce the incidence of severe toxicity in carriers without compromising on chemotherapy efficacy [5,6,7,8]. However, pre-emptive genotyping is not always beneficial [9].

An obvious limitation of the current genotype-based approach is that the four recommended *DPYD* variants are relatively rare and may explain only a small part of severe FP-related toxicity. Consequently, there is ongoing research into other *DPYD* gene variants that could improve toxicity risk prediction [10,11,12]. While some studies have thoroughly investigated rare variants of the *DPYD* gene [13], the clinical applicability of this new knowledge remains uncertain. On the other hand, more common variants of the *DPYD* gene appear to be promising markers for improving the prediction of FP-related toxicity. For example, alleles *4 (c.1601G>A, rs1801158), *6 (c.2194G>A, rs1801160), *9A (c.85T>C, rs1801265), and c.496A>G (rs2297595) of the *DPYD* gene have been associated with an increased risk of FP-related toxicity in several studies [10,14,15,16,17]. However, the impact and clinical validity of these and other alleles on FP-related toxicity requires further investigation, as evidence is currently insufficient or contradictory [18].

A promising approach to enhance the predictive value of *DPYD* variants is analyzing the polymorphism of the *MIR27A* gene, which encodes the microRNA miR-27a. This microRNA regulates DPD activity at the post-transcriptional level by inhibiting the expression of *DPYD* mRNA [19,20,21]. The variant allele rs895819 (A>G) in the *MIR27A* gene is associated with an increased expression of the *MIR27A* gene [19]. This variant has been shown to significantly improve the accuracy of toxicity prediction in carriers of the four recommended *DPYD* gene variants [22]. The combination of a high-risk *DPYD* gene variant and the minor G allele of rs895819 in the *MIR27A* gene significantly increased the risk of FP-related toxicity [22,23]. Moreover, patients with *DPYD* risk variants who received appropriate genotype-specific dose reductions remained at increased risk of fluoropyrimidine toxicity if they carried rs895819 in the *MIR27A* gene [21]. However, the effect of the *MIR27A* gene polymorphism on FP-related toxicity has primarily been studied in conjunction with the carriage of the four recommended *DPYD* variants [21,22,23]. We hypothesized that similar analyses might be useful for understanding the functional role and potential clinical utility of other common *DPYD* variants. Our research aimed to investigate whether the rs895819 variant in the *MIR27A* gene modifies the effect of five *DPYD* variants (c.1129-5923C>G, c.2194G>A, c.1601G>A, c.496A>G, c.85T>C) on the risk of FP-related toxicity.

## 2. Results

### 2.1. Characteristics of Studied Population

The prospective observational study included 370 patients with gastrointestinal tumors who received chemotherapy containing FPs (Table 1). Of these, 55 patients (14.9%) were treated with capecitabine-based regimens.

This study collected data on the incidence of overall severe toxicity (grade ≥ 3), neutropenia (grade ≥ 3), and asthenia (grade ≥ 2), which were each analyzed separately. Toxicity was assessed at two endpoints: (1) after the first course (early toxicity) and (2) after 4 courses, including all toxicities during courses 1–4. The choice of courses 1 to 4 as a secondary endpoint was based on the assumption that the majority of patients have first follow-up examination after 4 courses of therapy. Thus, the majority of patients (*n* = 333) received at least four courses of FP-containing chemotherapy. This selection of endpoints allows for the assessment of early toxicity as well as the cumulative impact of chemotherapy on the development of toxicity while reducing attrition bias or the influence of varying chemotherapy courses among patients. Thirty-seven patients received fewer than four courses of FPs due to disease progression, a change in therapy, or intolerable toxicity. For these patients, when assessing toxicity in courses 1–4, cases of toxicity were recorded if they occurred while receiving FPs. Otherwise, the data were considered not available (NA).

The development of toxicity was as follows: after the first course of FP-containing therapy, overall severe toxicity was observed in 62 patients (16.8%), including neutropenia in 27 (7.3%) and asthenia in 52 (14%). During courses 1–4, overall severe toxicity was observed in 148 of 352 patients (42%), neutropenia in 73 of 328 (22.3%), and asthenia in 83 of 328 (25.3%).

### 2.2. Genotypes of Studied Population

The genotypes were successfully determined in 367 patients (Table 2). The genotypes of three samples could not be determined due to insufficient DNA quality. The genotyping results for all markers were found to be in accordance with the Hardy–Weinberg equilibrium.

### 2.3. Identification of Clinical and Anthropometric Factors Influencing Toxicity

For each type of toxicity, we identified which clinical and anthropometric factors influenced its occurrence to further use significant (*p*-value < 0.05) variables as covariates in analyzing the influence of genetic variants on the risk of toxicity. The effects of sex, age, weight, body mass index (BMI), comorbidities, Eastern Cooperative Oncology Group (ECOG) performance status, and concomitant use of taxanes, oxaliplatin, irinotecan, and targeted therapy were assessed in a univariate analysis using logistic regression.

Regarding the first course toxicity endpoint, the co-administration of taxanes was a significant predictor of overall severe toxicity; significant predictors of neutropenia were the comorbidity and co-administration of taxanes; significant predictors of asthenia included sex (female), ECOG status, and weight (Table S1). Regarding adverse events after four courses of FP-containing chemotherapy, significant predictors of overall severe toxicity included sex (female), ECOG status, and the co-administration of irinotecan or taxanes; for neutropenia, significant factors were weight and co-administration of irinotecan or taxanes; for asthenia, predictors included sex (female), ECOG status, weight, age, and comorbidity (Table S1). Thus, these variables were used as covariates in multivariate logistic regression analysis to assess the role of genetic variants in the occurrence of these toxicities.

### 2.4. The Association of Genetic Markers with FP-Related Toxicity

We analyzed the impact of the studied genetic variants on FP-related toxicity using a dominant model, where carriers of variant alleles were compared with wild-type (wt) homozygotes. The modifying effect of the *MIR27A* gene polymorphism on the risk of toxicity in carriers of each studied *DPYD* gene variant was also analyzed. Carriers of *DPYD* gene variants were categorized into those who had the variant allele (G) of the *MIR27A* gene (*MIR27A^+^*) and those who did not (*MIR27A^−^*), and comparisons were made with wt-homozygotes. If the statistical significance of the *DPYD* allele varied depending on the presence of the *MIR27A* variant, interaction analysis was conducted.

All analyses were performed using multivariate logistic regression, incorporating significant clinical and anthropometric predictors (Table S1) as covariates. Consequently, the resulting odds ratios (ORs) and *p*-values were adjusted for risk factors associated with the corresponding types of toxicity.

#### 2.4.1. The Association of Genetic Markers with Early FP-Related Toxicity

The results of the analysis of genetic markers with early FP-related toxicity are presented in Table 3.

The DPYD*6 (c.2194G>A) was identified as a significant risk factor for neutropenia (OR 2.9, 95% CI 1.04–7.4, *p* = 0.031) with an increased risk specifically observed in those patients carrying both DPYD*6 and a variant allele of the *MIR27A* (*6 and *MIR27A^+^*) (OR 4.2, 95% CI 1.37–11.63, *p* = 0.008). Whereas, in carriers of the DPYD*6 and the wild-type *MIR27A* (*6 and *MIR27A^−^*), the risk was not increased. The interaction between DPYD*6 and *MIR27A* did not reach statistical significance (OR 9.19, 95% CI 0.96–219.95, *p* = 0.085).

The *DPYD* c.496G allele emerged as a risk factor for neutropenia only when combined with the *MIR27A* variant (496G and *MIR27A^+^*) (OR 3.15, 95% CI 1.05–8.51, *p* = 0.029). A statistically significant interaction between c.496G and *MIR27A* was observed (OR 6.35, 95% CI 1.09–43.39, *p* = 0.046), indicating that the co-presence of these variants significantly enhances the risk of neutropenia.

Other genetic markers included in the study showed no association with early FP-related toxicity.

#### 2.4.2. The Association of Genetic Markers with FP-Related Toxicity after 4 Courses

The analysis of genetic factors during the first four courses of FP-containing chemotherapy is summarized in Table 4.

DPYD*6 carriers showed an increased risk of overall severe toxicity (OR 2.12, 95% CI 1.07–4.27, *p* = 0.032) with a significant risk in *6 and *MIR27A^+^* carriers (OR 2.51, 95% CI 1.12–5.83, *p* = 0.027) but not in *6 and *MIR27A^−^* (*p* = 0.509). However, the interaction between DPYD*6 and *MIR27A* was not statistically significant for overall toxicity (OR 1.91, 95% CI 0.44–8.28, *p* = 0.38). For neutropenia, while DPYD*6 did not have a significant effect independently (*p* = 0.094), the risk was increased in *6 and *MIR27A^+^* carriers (OR 3.4, 95% CI 1.38–8.26, *p* = 0.007). A statistically significant interaction between DPYD*6 and *MIR27A* was observed (OR 7.35, 95% CI 1.28–61.53, *p* = 0.037), indicating that the co-presence of these variants significantly increases the risk of neutropenia. The risk of asthenia was increased for all DPYD*6 carriers (OR 3.16, 95% CI 1.44–6.92, *p* = 0.004), including both *6 and *MIR27A^−^* (OR 3.67, 95% CI 1.04–12.8, *p* = 0.039) and *6 and *MIR27A^+^* (OR 2.91, 95% CI 1.11–7.47, *p* = 0.026).

DPYD*4 was associated with the risk of neutropenia (OR 4.07, 95% CI 1.07–14.84, *p* = 0.033), and this risk was significant only in *4 and *MIR27A^+^* (OR 6.51, 95% CI 1.16–36.77, *p* = 0.026) but not in *4 and *MIR27A^−^* carriers (*p* = 0.443). The interaction between DPYD*4 and *MIR27A* was not statistically significant (*p* = 0.46).

No other genetic markers were associated with FP-related toxicity during the first four courses.

## 3. Discussion

Current guidelines for pharmacogenetic testing recommend the identification of four allelic variants of the *DPYD* gene (*2A—rs3918290, *13—rs55886062, —2846A>T—rs67376798, and —1236G>A/HapB3—rs56038477) before prescribing FPs [2,3,4]. For carriers, a dosage reduction or avoidance of these drugs is recommended. However, it is clear that these variants, due to their low frequency, cannot explain all cases of severe toxicity, even those mediated by a genetically determined decrease in DPD activity.

We found it encouraging that rs895819 in the *MIR27A* gene significantly improves the predictive value of high-risk *DPYD* variants for identifying patients at risk of severe FP-related toxicity [21,22,23]. Although these studies mainly included the four recommended *DPYD* variants, the article by Meulendijks et al. [23] also included the DPYD*4 allele. We hypothesized that a similar approach could enhance the significance and reproducibility for other variants in the *DPYD* gene that affect DPD activity. In the present study, we examined the association of five common variants in the *DPYD* gene with severe FP-related toxicity in patients with gastrointestinal tumors. Assuming that the effect of these genetic markers may depend on the status of the *MIR27A* gene, we also analyzed the studied alleles depending on the status of the *MIR27A* gene: without or together with the variant *MIR27A* allele (*MIR27A^−^* and *MIR27A^+^*, respectively).

The most pronounced role as a genetic risk factor for severe FP-related toxicity in our study was demonstrated by the DPYD*6 allele. This allele has been associated with various types of FP-related toxicity in numerous studies [10,14,15,16,24,25,26,27]. Although some researchers have reported negative results [28,29,30,31,32], a relatively recent meta-analysis confirmed the significance of this allele in European patients [33]. Our data suggest that the risk effect of DPYD*6 on FP-related toxicity may be modified by the presence of the *MIR27A* rs895819 variant, although this interaction varies across different types of toxicity. We observed an association of DPYD*6 with neutropenia at both endpoints (at course 1 and during courses 1–4), and the overall severe toxicity during courses 1–4 was found, specifically in patients carrying the *MIR27A* variant, but not in those without it. Direct confirmation of the interaction between these variants was obtained for the risk of neutropenia in the first four courses of FP-containing chemotherapy. On the other hand, the risk of asthenia (grade ≥ 2) in courses 1–4 was increased in all carriers of the DPYD*6 allele regardless of their *MIR27A* status. Further studies in larger cohorts are necessary to clarify the clinical relevance of the interaction between DPYD*6 and *MIR27A*. We suppose that DPYD*6 is indeed a promising marker for predicting FP-related toxicity and that testing for *MIR27A* could enhance its predictive value.

Another variant that showed an association with toxicity in our study was c.496G. The previously published results were controversial: while some studies described c.496G as a risk factor [16,17,27,30,34], others reported its protective effect [35], and several presented negative results [10,15,28,32,36,37]. We found that the risk of neutropenia in carriers of this allele is statistically significantly associated with the co-presence of the *MIR27A* variant. In patients with both 496G and *MIR27A^+^* alleles, the risk of neutropenia in the first cycle was increased, whereas for c.496G and *MIR27A^−^* patients, no association was noted, as well as in the overall group of c.496G carriers. Therefore, the interaction with *MIR27A* status may provide a more accurate assessment of the association of the c.496G variant with FP-related toxicity.

Additionally, neutropenia in courses 1–4 was associated with the DPYD*4 allele, and its effect was evident across the entire carrier group, as well as for patients with *4 and *MIR27A^+^*, but not for those with *4 and *MIR27A^−^*. However, in our study, the interaction of these variants was not significant, which might be explained by the insufficient sample size. Interestingly, a meta-analysis by Meulendijks et al. [23] showed that in patients with DPYD*4 (c.1601G>A), *MIR27A* variants were associated with the risk of severe FP-associated toxicity, and our results are consistent with these observations. It is worth noting that despite contradictory data, DPYD*4 is already used in preemptive testing in some studies [38], although data on its role are contradictory [39].

Other genetic markers included in our study, such as HapB3 and *9A in the *DPYD* gene, showed no association with severe FP-related toxicity. We examined the HapB3 variant, which is included in pharmacogenetic guidelines because they recommend an FP dose reduction only for homozygotes, which is extremely rare [2,3]. In a meta-analysis by Meulendijks et al., carriers of HapB3 were shown to have an increased risk of severe toxicity when also carrying the *MIR27A* variant [23]. However, we did not find an association of HapB3 with severe toxicity even when considering the *MIR27A* status.

The association of the DPYD*9A allele with FP-related toxicity is quite controversial according to published data: there is evidence of a risk [17,29], a protective effect [10,40], and no effect [16,27,28,30,41]. The analysis of this variant is complicated by the fact that it is linked to other SNPs, particularly c.496A>G, and there is evidence of the influence of the haplotype on enzyme activity and, consequently, the risk of toxicity [40,42,43]. We did not perform haplotype analysis in our study.

In our study, rs895819 in the *MIR27A* gene alone had no effect on the risk of FP-related toxicity but did show a modifying effect for some *DPYD* variants. We included only rs895819 in the *MIR27A* gene in our study, while a number of other studies also included rs11671784. However, rs11671784 was found to be less significant, occurs at a lower frequency [21,22,23], and is in strong linkage disequilibrium with rs895819 [23]. Therefore, we decided not to include it in our study.

The role of *MIR27A* variants has differed between studies. Amstutz et al. [22] reported a protective effect in patients without *DPYD* risk variants and an increased risk in carriers, but a subsequent meta-analysis by Meulendijks et al. showed an association with risk in carriers and in the overall population but no association in *DPYD* wild-type patients [23]. According to Falvella et al., severe side effects of FPs were associated with homozygous rs895819 [34], whereas Medwid et al. and Ragia et al. showed an increased risk in heterozygotes [21,44].

Thus, the data already published and obtained in the present study indicate that the variant allele rs895819 may have a risk effect that is more pronounced in carriers of dysfunctional *DPYD* variants. These results are logically consistent with the mechanisms underlying the influence of miR-27a on DPD activity. It is known that miR-27a targets the 3′-untranslated region of *DPYD* mRNA (this gene is one of its targets), inhibiting its translation [19,20,21,45]. MiR-27a expression has been found to be inversely correlated with *DPYD* mRNA levels and DPD activity [19]. The variant G allele of rs895819 leads to an increased expression of the *MIR27A* gene and, consequently, a decrease in DPD enzyme activity [19].

This study has some limitations. We selected *DPYD* gene variants based on a narrative review of the literature, but the testing of an expanded range of genetic markers seems to be reasonable in the future larger studies. No preliminary calculation of sample sizes was carried out, which was primarily because of the varying frequencies of minor alleles of the studied markers and the unknown effect size for most of them. When planning further validation studies, it is necessary to estimate sample sizes in advance to ensure the required statistical power. This study included only Caucasians, but further research is needed to confirm these findings in larger and more diverse populations.

## 4. Materials and Methods

### 4.1. Patients

This prospective observational study included Caucasian patients with gastrointestinal tumors (gastric cancer, colorectal cancer, pancreatic cancer, small intestine cancer, esophageal cancer, and liver cancer), who received FP-based chemotherapy at the City Clinical Hospital named after S.S. Yudin of the Moscow Health Department (Chemotherapy Department No. 1) from October 2020 to May 2022. Exclusion criteria were age younger than 18 years and a history of previous anticancer chemotherapy or radiotherapy. All the patients signed an informed consent to participate in the study. The study was conducted in accordance with the Declaration of Helsinki and approved by the Local Ethics Committee of the Russian Medical Academy of Continuous Professional Education of the Ministry of Healthcare, Moscow, Russian Federation (protocol No. 9, date of approval 7 July 2020). The collection of blood samples and prospective follow-up of the patients was carried out under the Russian Science Foundation project No. 20-75-10158. All the patients underwent pharmacogenetic testing of the DPYD*2A allele before the 1st course of chemotherapy. Carriers of the DPYD*2A allele were not included in this study.

Toxicity was assessed prospectively according to the National Cancer Institute Common Terminology Criteria for Adverse Events (CTCAE) v5.0.

### 4.2. Genotyping

DNA was isolated from venous blood samples collected in EDTA-containing tubes using QIamp DNA Mini kits (Qiagen, Hilden, Germany). The concentration of DNA was assessed using a Nanodrop ND-1000 spectrophotometer (Thermo Fisher Scientific, Waltham, MA, USA).

The study included five variants in the *DPYD* gene: c.1129-5923C>G (rs75017182, HapB3), c.2194G>A (rs1801160, *6), c.1601G>A (rs1801158, *4), c.496A>G (rs2297595), and c.85T>C (rs1801265, *9A) as well as rs895819 (A>G) in the *MIR27A* gene. Genotypes were determined by high-resolution melting analysis (HRM) using a LightCycler 96 (Roche, Basel, Switzerland). The composition of the reaction and the amplification program, as well as the sequences of the primers used, are provided in the Supplementary Materials (Supplementary Methods, Table S2).

### 4.3. Statistical Analysis

Statistical analysis was performed using R version 4.3.1 (R Foundation for Statistical Computing, Vienna, Austria). The influence of clinical and anthropometric factors on toxicity was assessed using univariate logistic regression. The effect of genetic variants on toxicity was tested using multivariate logistic regression with statistically significant clinical and anthropometric factors used as covariates. An exact test for Hardy–Weinberg equilibrium was performed using the “HardyWeinberg” package (version 1.7.8). Differences were considered statistically significant when the *p*-value was below 0.05. No adjustment for multiple comparisons was applied.

## 5. Conclusions

Our findings suggest that the risk of FP-related toxicity in *DPYD* *4, *6, and c.496G carriers may depend on the rs895819 *MIR27A* variant. Thus, we demonstrated that an approach accounting for the modifying effect of the *MIR27A* gene polymorphism is promising when studying the association of *DPYD* variants with FP-related toxicity. We believe that this strategy may be useful in studying other genetic markers in the *DPYD* gene in future research, ultimately improving the accuracy of existing approaches to predicting toxicity based on the patient’s genotype and enhancing the safety of FP-containing chemotherapy.

## Figures and Tables

**Table 1:** Baseline characteristics of patients with gastrointestinal tumors.

Characteristic	Value (*n* = 370)
Sex (female), *n* (%)	160 (43.2%)
Age, mean ± sd	64.53 ± 10.31
Weight, mean ± sd	74.05 ± 16.41
BMI, mean ± sd	26.17 ± 9.14
Tumor localization, *n* (%):	
Colon	138 (37.3%)
Stomach	125 (33.8%)
Rectum	50 (13.5%)
Pancreas	44 (11.9%)
Esophagus	9 (2.4%)
Small intestine	2 (0.5%)
Biliary tract	2 (0.6%)
Chemotherapy type, *n* (%):	
line 1	191 (51.6%)
adjuvant	91 (24.6%)
neoadjuvant	88 (23.8%)
Stage at diagnosis, *n* (%):	
1	5 (1.4%)
2	55 (14.9%)
3	133 (35.9%)
4	177 (47.8%)
Smoking, *n* (%)	103 (27.8%)
ECOG, *n* (%):	
0	106 (28.6%)
1	237 (64.1%)
2	27 (7.3%)
Target therapy, *n* (%):	
No	305 (82.5%)
Yes, including:	65 (17.5%)
Bevacizumab	39 (10.5%)
Cetuximab	11 (3%)
Panitumumab	11 (3%)
Trastuzumab	4 (1.1%)
Irinotecan, *n* (%)	51 (13.8%)
Oxaliplatin, *n* (%)	350 (94.6%)
Taxanes, *n* (%)	47 (12.7%)

BMI—body mass index; ECOG—Eastern Cooperative Oncology Group performance status; SD—standard deviation.

**Table 2:** *DPYD* and *MIR27A* genotyping in patients with gastrointestinal tumors.

rs ID (Allele)	Nucleotide Change	Wild-Type Homozygotes, *n* (%)	Heterozygotes *n* (%)	Minor Allele Homozygotes *n* (%)	MAF	HWE *p*-Value
*DPYD*						
rs75017182 (HapB3)	c.1129-5923C>G	353 (96.2%)	14 (3.8%)	0 (0%)	1.9%	1
rs1801160 (*6)	c.2194G>A	324 (88.3%)	41 (11.2%)	2 (0.5%)	6.1%	0.636
rs1801158 (*4)	c.1601G>A	356 (97.0%)	11 (3.0%)	0 (0%)	1.5%	1
rs2297595	c.496A>G	289 (78.8%)	76 (20.7%)	2 (0.5%)	10.9%	0.285
rs1801265 (*9A)	c.85T>C	208 (56.7%)	129 (35.1%)	30 (8.2%)	19.1%	0.133
*MIR27A*						
rs895819	A>G	154 (42%)	171 (46.6%)	42 (11.4%)	34.7%	0.646

MAF—Minor Allele Frequency; HWE—Hardy–Weinberg equilibrium.

**Table 3:** Risk of early fluoropyrimidine-associated toxicity according to *DPYD* and *MIR27A* status.

Genetic Variant	Genotype	Overall Severe Toxicity (Adjusted by Taxanes)			Neutropenia Grade ≥ 3 (Adjusted by Comorbidity, Taxanes)			Asthenia Grade ≥ 2 (Adjusted by Sex, ECOG, Weight)		
		No/Yes	OR (95% CI)	*p*-Value	No/Yes	OR (95% CI)	*p*-Value	No/Yes	OR (95% CI)	*p*-Value
c.1129-5923 C>G (HapB3)	wt	294/59	-	-	327/26	-	-	302/50	-	-
	HapB3	11/3	1.43 (0.31–4.79)	0.596	13/1	1.1 (0.06–6.17)	0.927	12/2	1.21 (0.18–4.98)	0.812
	HapB3 and *MIR27A^−^*	5/3	3.02 (0.60–12.80)	0.142	7/1	1.82 (0.09–11.50)	0.591	7/1	0.75 (0.04–4.64)	0.796
	HapB3 and *MIR27A^+^*	6/0	NA	0.988	6/0	NA	0.989	5/1	2.65 (0.13–19.76)	0.404
c.2194G>A (*6)	wt	274/50	-	-	304/20	-	-	281/42	-	-
	*6	31/12	1.98 (0.92–4.06)	0.070	36/7	2.9 (1.04–7.40)	0.031	33/10	2.23 (0.95–4.9)	0.053
	*6 and *MIR27A^−^*	10/3	1.46 (0.31–5.07)	0.580	12/1	0.93 (0.05–5.66)	0.944	9/4	3.37 (0.85–11.46)	0.060
	*6 and *MIR27A^+^*	21/9	2.24 (0.92–5.08)	0.062	24/6	4.2 (1.37–11.63)	0.008	24/6	1.8 (0.62–4.62)	0.243
c.1601G>A (*4)	wt	296/60		-	330/26	-	-	306/49	-	-
	*4	9/2	1.24 (0.19–5.02)	0.785	10/1	2.01 (0.11–11.8)	0.520	8/3	2.44 (0.51–9.02)	0.208
	*4 and *MIR27A^−^*	5/0	NA	0.983	5/0	NA	0.990	3/2	4 (0.5–25.63)	0.142
	*4 and *MIR27A^+^*	4/2	2.8 (0.38–14.76)	0.243	5/1	4.61 (0.23–31.79)	0.179	5/1	1.4 (0.07–9.19)	0.765
c.496A>G	wt	240/49	-	-	271/18	-	-	249/39	-	-
	496G	65/13	0.95 (0.47–1.82)	0.874	69/9	1.9 (0.77–4.44)	0.145	65/13	1.24 (0.59–2.47)	0.770
	496G and *MIR27A^−^*	37/5	0.64 (0.21–1.6)	0.382	39/3	1.04 (0.23–3.38)	0.959	36/6	1.03 (0.36–2.51)	0.973
	496G and *MIR27A^+^*	26/8	1.35 (0.54–3.03)	0.495	30/6	3.15 (1.05–8.51)	0.029	29/7	1.52 (0.56–3.7)	0.611
c.85T>C (*9A)	wt	171/37	-	-	193/15	-	-	177/31	-	-
	*9A	134/25	0.89 (0.51–1.54)	0.678	147/12	1.35 (0.59–3.07)	0.471	137/21	0.98 (0.53–1.81)	0.947
	*9A and *MIR27A^−^*	65/10	0.73 (0.33–1.51)	0.419	70/5	1.09 (0.33–3.07)	0.875	62/12	1.11 (0.51–2.30)	0.778
	*9A and *MIR27A^+^*	69/15	1.03 (0.52–1.96)	0.927	77/7	1.6 (0.59–4.08)	0.336	75/9	0.85 (0.37–1.84)	0.693
*MIR27A* (A>G)	wt	130/24	-	-	141/13	-	-	127/26	-	-
	*MIR27A^+^*	175/38	1.19 (0.68–2.10)	0.548	199/14	0.84 (0.38–1.89)	0.662	187/26	0.75 (0.41–1.38)	0.357

**Table 4:** Risk of fluoropyrimidine-associated toxicity according to *DPYD* and *MIR27A* status during 1–4 courses.

Genetic Variant	Genotype	Overall Severe Toxicity (Adjusted by Sex, ECOG, Irinotecan, Taxanes)			Neutropenia Grade ≥ 3 (Adjusted by Irinotecan, Taxanes, Weight)			Asthenia Grade ≥ 2 (Adjusted by Sex, ECOG, Weight, Age, Comorbidity)		
		No/Yes	OR (95% CI)	*p*-Value	No/Yes	OR (95% CI)	*p*-Value	No/Yes	OR (95% CI)	*p*-Value
c.1129-5923 C>G (HapB3)	wt	194/144	-	-	244/72	-	-	234/82	-	-
	HapB3	10/4	0.69 (0.18–2.27)	0.563	11/1	0.40 (0.02–2.24)	0.398	11/1	0.23 (0.01–1.38)	0.182
	HapB3 and *MIR27A^−^*	5/3	0.82 (0.15–3.68)	0.801	6/1	0.65 (0.03–4.19)	0.703	6/1	0.34 (0.02–2.43)	0.358
	HapB3 and *MIR27A^+^*	5/1	0.50 (0.03–3.37)	0.541	5/0	NA	0.983	5/0	NA	0.988
c.2194G>A (*6)	wt	186/124	-	-	229/60	-	-	224/67	-	-
	*6	18/24	2.12 (1.07–4.27)	0.032	26/13	1.92 (0.87–4.08)	0.094	21/16	3.16 (1.44–6.92)	0.004
	*6 and *MIR27A^−^*	6/7	1.48 (0.46–4.87)	0.509	11/2	0.51 (0.07–2.08)	0.401	7/6	3.67 (1.04–12.8)	0.039
	*6 and *MIR27A^+^*	12/17	2.51 (1.12–5.83)	0.027	15/11	3.40 (1.38–8.26)	0.007	14/10	2.91 (1.11–7.47)	0.026
c.1601G>A (*4)	wt	199/142	-	-	249/68	-	-	239/78	-	-
	*4	5/6	1.77 (0.51–6.38)	0.363	6/5	4.07 (1.07–14.84)	0.033	6/5	2.39 (0.64–8.55)	0.178
	*4 and *MIR27A^−^*	3/2	0.76 (0.10–4.82)	0.772	3/2	2.15 (0.25–15.17)	0.443	3/2	2.23 (0.27–15.19)	0.411
	*4 and *MIR27A^+^*	2/4	3.59 (0.68–26.46)	0.148	3/3	6.51 (1.16–36.77)	0.026	3/3	2.51 (0.44–14.42)	0.28
c.496A>G	wt	162/116	-	-	204/55	-	-	198/63	-	-
	496G	42/32	0.86 (0.49–1.49)	0.592	51/18	1.02 (0.52–1.93)	0.952	47/20	1.21 (0.63–2.27)	0.555
	496G and *MIR27A^−^*	25/15	0.64 (0.30–1.31)	0.233	29/8	0.75 (0.29–1.75)	0.524	28/10	1.05 (0.44–2.32)	0.912
	496G and *MIR27A^+^*	17/17	1.19 (0.56–2.53)	0.645	22/10	1.39 (0.58–3.17)	0.441	19/10	1.46 (0.58–3.49)	0.407
c.85T>C (*9A)	wt	106/92	-	-	141/41	-	-	136/48	-	-
	*9A	98/56	0.64 (0.41–1.01)	0.056	114/32	0.94 (0.54–1.62)	0.824	109/35	0.82 (0.48–1.41)	0.476
	*9A and *MIR27A^−^*	46/27	0.63 (0.35–1.12)	0.118	55/13	0.77 (0.36–1.55)	0.473	50/19	0.94 (0.48–1.81)	0.859
	*9A and *MIR27A^+^*	52/29	0.66 (0.37–1.14)	0.139	59/19	1.10 (0.57–2.08)	0.766	59/16	0.71 (0.35–1.39)	0.33
*MIR27A* (A>G)	wt	86/66	-	-	110/30	-	-	102/41	-	-
	*MIR27A^+^*	118/82	1.0 (0.64–1.56)	0.990	145/43	1.20 (0.69–2.11)	0.515	143/42	0.84 (0.49–1.42)	0.505

## Data Availability

The data presented in this study are available from the corresponding author upon reasonable request.

## Supplementary Materials

The supporting information can be downloaded at https://www.mdpi.com/article/10.3390/ijms25158503/s1.
