# Supplementary material for: MIR27A Gene Polymorphism Modifies the Effect of Common DPYD Gene Variants on Severe Toxicity in Patients with Gastrointestinal Tumors Treated with Fluoropyrimidine-Based Anticancer Therapy
Source: Int J Mol Sci. 2024 Aug 4;25(15):8503. doi: 10.3390/ijms25158503 (PMC11313059; doi:10.3390/ijms25158503)
Supplement: Supplementary file 1 [file ijms-25-08503-s001.zip › Supplementary Methods.pdf]

Supplementary Methods

Genotypes of DNA samples were determined using High Resolution Melting analysis (HRM). The reaction mixture with a volume of 20 µl contained 1×PCR buffer (Synthol, Moscow, Russia), 1.5 mM MgCl2, 1×Eva488 (Lumiprobe RUS Ltd., Moscow, Russia), 50 µM of each dNTP (Silex, Moscow, Russia), 300 nM of both direct and reverse primers (Lumiprobe RUS Ltd., Moscow, Russia), 1 unit of SynTaq polymerase (Synthol, Moscow, Russia), and 5 – 50 ng of DNA. The primers used for analyzing each genetic marker are listed in Table 2. The analysis was conducted using a LightCycler 96 amplifier (Roche, Basel, Switzerland) following this program: 94°C for 4 min, followed by 35 cycles of 94°C for 20 s, 62°C for 15 s, and 72°C for 15 s, cooling to 40°C, and then melting from 65 to 95°C at 15 readings per 1°C. Detection was performed using the ResoLight channel. LightCycler 96 software version 1.1 (Roche, Basel, Switzerland) was employed to analyze the results. The genotypes of control samples were determined by Sanger sequencing.

Table S2. Primer sequences

Gene	rs ID (allele)	Nucleotide change	Primer direction	Sequence 5'-3'
DPYD	rs75017182 (*HapB3*)	c.1129-5923C>G	Forward	TGACAAATCAGGTTGTCACTTTCT
			Reverse	TTCACTCAGCATCAGCCACA
	rs1801160 (**6*)	c.2194G>A	Forward	GGTGTGCCATCAGATTTTAATCCC
			Reverse	CATTTTTCTGGGATGTGAGGGTTTG
	rs1801158 (**4*)	c.1601G>A	Forward	ATAAACTTCAATCCGGCCATTTCTACA
			Reverse	TGTATTTTGCAGTCACAATATGGAGC
	rs2297595	c.496A>G	Forward	CAGGCCCAGCACCAAAAAGA
			Reverse	CCATGACAATTGATTTCCCCGT
	rs45589337	c.775A>G	Forward	TGTAGCCTTTTTCTTTCAAAGTGCT
			Reverse	ATTTTAATTAATCATATGCCAAATTTTCTTATGC
	rs1801265 (**9A*)	c.85T>C	Forward	AATTTCTTGGCCCGAAGTGGAAC
			Reverse	TGCTGTCTTTAGAGTATCCTGGCT
MIR27A	rs895819	40A>G	Forward	AGGCCAGAGGAGGTGAGGG
			Reverse	TAGCTGCTTGTGAGCAGGGT
