# Supplementary material for: MIR27A Gene Polymorphism Modifies the Effect of Common DPYD Gene Variants on Severe Toxicity in Patients with Gastrointestinal Tumors Treated with Fluoropyrimidine-Based Anticancer Therapy
Source: Int J Mol Sci. 2024 Aug 4;25(15):8503. doi: 10.3390/ijms25158503 (PMC11313059; doi:10.3390/ijms25158503)
Supplement: Supplementary file 1 [file ijms-25-08503-s001.zip › Table S1.pdf]

Table S1. Univariate logistic regression analysis of the influence of clinical factors on the risk of toxicity in patients taking fluoropyrimidines

Toxicity type	Predictor	1 course		1 – 4 courses	
		OR (95%CI)	p-value	OR (95%CI)	p-value
**Overall severe toxicity (grade ≥3)**	Sex (male)	0,63 (0,36 - 1,08)	0,095	0,62 (0,4 - 0,95)	**0,028**
	Age	1 (0,97 - 1,03)	0,974	0,99 (0,97 - 1,01)	0,525
	Comorbidity	0,82 (0,44 - 1,59)	0,535	0,64 (0,38 - 1,06)	0,085
	ECOG	1,51 (0,92 - 2,49)	0,106	1,64 (1,11 - 2,43)	**0,013**
	Target therapy	0,76 (0,33 - 1,55)	0,471	0,83 (0,48 - 1,44)	0,517
	Oxaliplatin	0,8 (0,28 - 2,88)	0,704	0,97 (0,33 - 2,99)	0,95
	Irinotecan	0,62 (0,23 - 1,42)	0,296	1,94 (1,05 - 3,63)	**0,034**
	Taxanes	2,19 (1,05 - 4,37)	**0,031**	2,45 (1,29 - 4,77)	**0,007**
	Weight	0,98 (0,97 - 1)	0,076	0,99 (0,98 - 1,01)	0,275
	BMI	0,97 (0,91 - 1,01)	0,242	0,98 (0,95 - 1,01)	0,334
**Neutropenia (grade ≥3)**	Sex (male)	0,82 (0,37 - 1,82)	0,621	0,64 (0,38 - 1,08)	0,092
	Age	0,98 (0,94 - 1,02)	0,238	1 (0,98 - 1,03)	0,933
	Comorbidity	0,36 (0,16 - 0,82)	**0,013**	0,6 (0,34 - 1,11)	0,096
	ECOG	1,12 (0,55 - 2,27)	0,751	1,5 (0,92 - 2,48)	0,11
	Target therapy	0,17 (0,01 - 0,8)	0,081	0,59 (0,27 - 1,18)	0,159
	Oxaliplatin	0,7 (0,19 - 4,55)	0,643	1,3 (0,33 - 8,65)	0,742
	Irinotecan	1,45 (0,47 - 3,75)	0,473	3,1 (1,58 - 5,99)	**<0,001**
	Taxanes	4,09 (1,65 - 9,59)	**0,001**	2,38 (1,16 - 4,76)	**0,016**
	Weight	0,99 (0,96 - 1,01)	0,317	0,98 (0,97 - 1)	**0,04**
	BMI	0,97 (0,89 - 1,02)	0,392	0,96 (0,91 - 1,01)	0,137
**Astenia (grade ≥2)**	Sex (male)	0,47 (0,26 - 0,85)	**0,014**	0,36 (0,22 - 0,61)	**<0,001**
	Age	1,02 (0,99 - 1,05)	0,19	1,04 (1,01 - 1,06)	**0,01**

**Toxicity type**	**Predictor**	**1 course**		**1 – 4 courses**	
		**OR (95%CI)**	**p-value**	**OR (95%CI)**	**p-value**
	Comorbidity	1,35 (0,65 - 3,07)	0,448	2,08 (1,07 - 4,37)	**0,04**
	ECOG	2,72 (1,56 - 4,86)	**<0,001**	2,64 (1,62 - 4,45)	**<0,001**
	Target therapy	0,56 (0,21 - 1,29)	0,21	0,64 (0,31 - 1,24)	0,206
	Oxaliplatin	1,52 (0,42 - 9,75)	0,582	0,9 (0,25 - 4,18)	0,879
	Irinotecan	0,62 (0,21 - 1,51)	0,335	1,09 (0,51 - 2,17)	0,821
	Taxanes	1,57 (0,67 - 3,35)	0,269	1,14 (0,52 - 2,34)	0,733
	Weight	0,98 (0,96 - 1)	**0,04**	0,98 (0,96 - 1)	0,022
	BMI	0,95 (0,89 - 1)	0,106	1,01 (0,99 - 1,04)	0,333

BMI – body mass index; ECOG – Eastern Cooperative Oncology Group performance status; OR – odds ratio; CI – confidence interval
